# The interplay of domain-and life satisfaction in predicting life events

**DOI:** 10.1371/journal.pone.0238992

**Published:** 2020-09-17

**Authors:** Till Kaiser, Marie Hennecke, Maike Luhmann

**Affiliations:** 1 Ruhr-Universität Bochum, Bochum, Germany; 2 Universität Siegen, Siegen, Germany; Mälardalen University, SWEDEN

## Abstract

To better understand the occurrence of major changes in people´s lives like job changes or relocations, we test a model of motivational consequences of life and domain satisfaction using data of the German socio-economic panel study (SOEP) (waves 2005–2015; Ns between 2,201 and 28,720). We examined job and location changes as outcomes that people may actively initiate as a result of dissatisfaction with these domains. One of our results indicates that for similar levels of job satisfaction, individuals with higher levels of life satisfaction were more likely to report a subsequent job change, presumably because they possess necessary resources to actively initiate such a major life change. The patterns were similar for relocation satisfaction and subsequent relocation, but not all effects were significant. Generally, the effects of life satisfaction and domain satisfaction on life events were independent of affective well-being. Contrary to what we expected based on life-span theories, perceived control did not significantly moderate the tested mechanisms. These findings furthermore show that examining life satisfaction and domain satisfaction in isolation can lead to theoretically and empirically false conclusions. Contrary to previous research, high life satisfaction appears to not be a general driver for stability but rather should be seen as an indicator of resourcefulness that allows people to strive for changes in specific life domains.

## Introduction

Many people experience major changes in their life circumstances at some point in their lives. For some people, these major life changes just happen to them, with little control over whether and when they occur. Others, however, actively initiate major life changes. For example, they actively decide to relocate, change jobs, or enter or leave a relationship. There are many reasons for why people seek out major changes, but one motivating factor that appears to play a role in many of these kinds of decisions is that people are dissatisfied with their current status. For example, people are more likely to relocate if they are dissatisfied with their current housing situation [1–3], to change jobs if they dislike their current one [4–7], or to file for divorce if they are dissatisfied with their marriage [8, 9]. Put simply, people who are dissatisfied with a particular life domain are more likely to change something within this life domain. In addition, some studies have shown that one’s general life satisfaction prospectively predicts major life changes [10, 11], and that people low in life satisfaction reported a greater desire to change their life circumstances, underpinning the assumption that people self-initiate changes due to dissatisfaction [11].

Together, previous research has shown that both general life satisfaction and satisfaction with specific life domains (henceforth: domain satisfaction) may motivate people to seek major changes in their life circumstances. However, an important limitation of previous studies is that they examine the effects of life satisfaction or domain satisfaction, but a systematic comparison of both variables predicting life events is missing. This is problematic because interpreting the effect of one predictor without considering the effect of the other may lead to false conclusions [12]. For example, it is possible that people who change their jobs do so because they are dissatisfied with their job (effect of domain satisfaction; [2]), but it is also possible that they are driven by a more diffuse sense of general dissatisfaction that may have little to do with their current job (effect of general life satisfaction; [10]). Furthermore, both domain satisfaction and general life satisfaction might independently affect people’s likelihood to seek out major life changes. In sum, to understand how and when (dis)satisfaction may lead people to actively initiate major life changes, it is necessary to consider the joint effects of domain satisfaction and general life satisfaction on major life outcomes, something that has been neglected in this literature to date. Therefore, our paper is a first step to compare the effects of domain satisfaction and life satisfaction on life events.

We present a novel theoretical model on people’s satisfaction with a particular life domain and their satisfaction with life overall in predicting major life outcomes. We examine this model empirically with regard to major life events in two life domains: job change and relocation. These two life events are particularly suitable to test our theoretical model because they have consistently been shown to be influenced by both domain satisfaction and general life satisfaction in previous research [1–7, 10].

### The interrelation between life satisfaction and domain satisfaction

**Fig 1:**
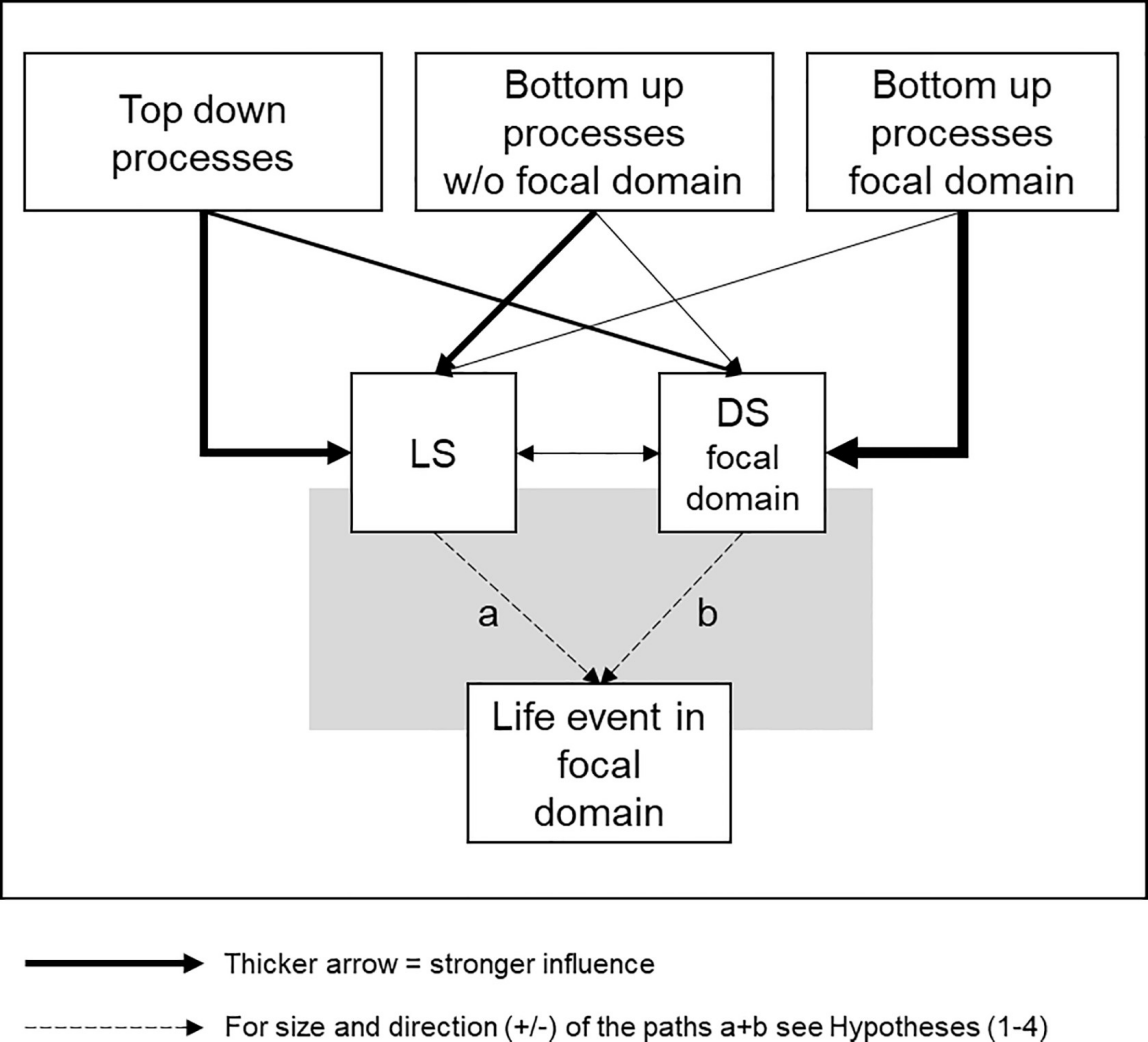
Working model on the interrelation between life satisfaction and domain satisfaction. Grey box highlights the pathways tested in the present article. (LS = Life satisfaction, DS = Domain satisfaction.

In our theoretical model (see Fig 1), we assume that satisfaction with a particular life domain (domain satisfaction) and satisfaction with one’s life overall (henceforth: life satisfaction) are affected by both bottom-up and top-down influences [13–16]. Bottom-up influences represent perceived discrepancies between current life circumstances and desired circumstances. Positive discrepancies (if current circumstances exceed desired circumstances) have positive effects on satisfaction; negative discrepancies (if current circumstances fall short of desired circumstances) have negative effects on satisfaction [13–15]. A negative discrepancy between people’s desired life circumstances and their actual life circumstances constitutes a bottom-up influence on satisfaction, which may then lead to a desire for change and the initiation of life events that can bring about such change to overcome low satisfaction [11]. Top-down influences are “sources of belief, affect, or disposition” [15] that affect the satisfaction judgments and are potentially independent of life circumstances [15, 16]. For instance, top-down influences include self-esteem and the personality traits extraversion and neuroticism, which are predictors of life satisfaction [17, 18].

Previous research has shown that bottom-up influences, for example changes within a particular domain (e.g., a raise at work), are more likely to affect a person´s satisfaction with the particular domain (e.g., work) than their life satisfaction [15]. In contrast, top-down influences such as personality traits are more likely to affect life satisfaction, even after controlling for satisfaction with particular domains [15, 19, 20]. Our theoretical model (Fig 1) integrates these assumptions on the different influences of bottom-up and top-down processes on domain satisfaction and life satisfaction. In addition, this model assumes that bottom-up influences in other domains (e.g. health) than the focal domain (e.g. housing) should generally have stronger influences on general life satisfaction than on satisfaction with the focal domain.

Applied to the life domains examined in our study, we assume that job characteristics should have a stronger impact on job satisfaction than on life satisfaction. Therefore, low job satisfaction should motivate people to change their jobs independently of their level of general life satisfaction. Similarly, we assume that housing circumstances should have a stronger impact on housing satisfaction than on life satisfaction, and low housing satisfaction should motivate people to relocate, independently of their level of general life satisfaction. Hence, we expect a negative effect of domain satisfaction on life events (Hypothesis 1, b < 0 in Fig 1), and we further expect that this effect remains negative if domain satisfaction and life satisfaction are analyzed simultaneously (Hypothesis 3).

With respect to life satisfaction, previous research has found that higher life satisfaction is associated with a lower likelihood of life events such as job change and relocation [10, 11]. We expect this to be the case in our study when examining life satisfaction in isolation (Hypothesis 2, a < 0 in Fig 1), because life satisfaction is partly determined by satisfaction in the focal domain. However, since life satisfaction is also determined by other bottom-up processes and top- down processes, we assume that the negative effect of life satisfaction on the life event in the focal domain should not be as strong as the effect of domain satisfaction on the life event.

### Three competing perspectives on the effect of life satisfaction on life events

As elaborated previously, we expect that lower life satisfaction predicts life events like job change and relocation (Hypothesis 2, Fig 1 a < 0). We were less certain, however, how the effect of life satisfaction changes after controlling for domain satisfaction. Controlling for domain satisfaction, the association between life satisfaction and relocation and job change might be zero (Hypothesis 4a, Fig 1 a = 0), negative (Hypothesis 4b, Fig 1 a < 0), or positive (Hypothesis 4c, Fig 1 a > 0). These competing hypotheses are best understood in the context of our model shown in Fig 1.

Hypothesis 4a (a = 0) assumes that there is only an effect of satisfaction with the focal domain (b) on events within this domain. Neither satisfaction with other life domains nor any other influences on life satisfaction such as personality or other top-down processes are relevant, and therefore, the effect of life satisfaction on the likelihood of the event becomes zero (a = 0) once domain satisfaction is controlled (b). In this version, the influence of the focal domain erases the effects of all other factors.

Hypotheses 4b (a < 0) and 4c (a > 0) both assume that the likelihood of life events is additionally influenced by other factors through their effects on life satisfaction. Hypothesis 4b (a < 0) is based on the discrepancy perspective on life satisfaction, according to which low life satisfaction is the product of a perceived negative discrepancy between one’s desired and one’s actual life circumstances [14], and changes in life are seen as attempts to reduce this discrepancy [11]. This means that for similar levels of satisfaction with the focal domain, individuals with lower levels of life satisfaction should be *more* likely to initiate changes in this domain than individuals with higher levels of life satisfaction (a < 0) because they are generally more motivated to seek out changes. Applied to the domains of work and housing, this hypothesis specifically predicts that people with high levels of life satisfaction are less likely to relocate or change jobs, and people with low levels of life satisfaction are more likely to relocate or change jobs, independent of whether they are currently satisfied or dissatisfied with their housing or job.

Hypothesis 4c predicts that for similar levels of satisfaction with the focal domain, individuals with lower levels of life satisfaction should be *less* likely to initiate changes in this domain than individuals with higher levels of life satisfaction (a > 0). This hypothesis is derived from *conservation of resource theory* (COR) [21–23]. The basic assumption of COR is that individuals strive to gain, protect, and maintain resources. According to the resource investment principle of COR, people need to invest their resources to gain new ones and to protect and recover from resource losses. Assuming that life satisfaction is both reflective of having material and social resources and is a psychological resource in itself [15], this principle implies that, all else being equal, people with higher levels of life satisfaction are more likely to actively initiate life events because they possess the necessary resources. Hence, this theoretical perspective predicts that for constant levels of domain satisfaction, higher levels of life satisfaction are associated with an increased likelihood of job change and relocation (a > 0).

### Moderator effects

So far, the joint effects of life satisfaction and domain satisfaction were conceptualized in terms of additive main effects. However, it is possible that the magnitude of these main effects depends on other variables. In this paper, we focus on two sets of moderators.

First, we explore interaction effects between life satisfaction and domain satisfaction. Adding interaction effects allows us to test whether the effect of one of these predictors depends on the level of the other predictor. For example, it is possible that life satisfaction is only associated with the likelihood of specific life events if domain satisfaction is low, but not if it is high. We had no specific hypotheses about the existence and direction of these kinds of interaction effects, so these analyses should be considered exploratory.

Second, we examine perceived control as a possible moderator. Perceived control is a crucial construct to explain successful individual development through the life span [24]. It can be broadly defined as people’s belief in their ability to exert influence over and shape their own life circumstances [25–27]. People with high perceived control think that they have a strong impact on what happens in their lives, whereas people with low perceived control believe that events in their lives are determined by external sources like fate, luck, or powerful others [28].

Perceived control is of particular interest to explain the active initiation of life events because if “individuals perceive themselves as agentic in mastering developmental goals or tasks, they are encouraged to engage in striving for desirable goals and, in consequence, are more likely to experience success.” [24]. We therefore hypothesize that people with low domain satisfaction are more likely to initiate life events in the focal domain to overcome their low domain satisfaction if they perceive that they have control over their life circumstances (Hypothesis 5). Similarly, we hypothesize that the unique relationship between life satisfaction and life events (which may be positive or negative controlling for domain satisfaction, see above) is moderated by perceived control such that the relationship is stronger for higher levels of perceived control (Hypothesis 6).

### The present study

The present study contributes to previous research by examining the interplay of domain satisfaction and life satisfaction in predicting major life changes in the domains of work (job change) and housing (relocation). Based on the motivational consequences of life satisfaction model [11], theories on bottom-up and top-down influences [15] and conservation of resource theory [23] we examine partially competing hypotheses using data from the Socioeconomic Panel Study (SOEP).

For our analyses, we also took affective well-being into account, since affect plays a central role in many motivational theories [29, 30] and affective well-being forms together with cognitive well-being (life satisfaction and domain satisfaction) the construct of subjective well-being [16]. Affective well-being refers to people’s emotional experiences as reflected by how often they experience negative and positive affect. affective well-being and cognitive well-being are moderately correlated but are structurally und functionally distinct constructs with unique predictors and consequences [31, 32]. For instance, life circumstances and life events have been shown to be stronger related to cognitive well-being while personality was stronger correlated with affective well-being [33, 34]. Luhmann and Hennecke [11] also showed that while cognitive well-being was consistently predictive for people desire to change the effects of affective well-being were inconsistent. Due to these findings, we suggest that it is important to take into account affective well-being, but that it should not play a significant role in predicting future life events.

Using 12 waves (2004–2015) of the SOEP, we predicted the likelihood of relocation and job change in one wave by life satisfaction and domain satisfaction in the previous wave. To do so we used logistic multilevel models (person-year observations clustered within persons).

## Methods

### Sample

Data came from the German Socio-Economic Panel Study (SOEP). The SOEP is a representative annual household panel study that started in 1984 [35]. All data used in this manuscript have been collected in a manner consistent with ethical standards for the treatment of human subjects. The data was completely anonymized and de-identified before the authors had access to it. The analyses were based on unbalanced longitudinal datasets of the SOEP waves 2004 to 2015 that contain information on the individuals and their households (*N* = 2,037–28,778). The sample used to analyze the event of job change consisted of individuals who are employed only to ensure that we examined the event of job change within the labor market and not the event of job re-entry into the labor market. For a description of the variables used in the study (means, standard deviation, range, and scale), see Table 1.

**Table 1:** Sample description.

Variables	Mean /			
	Percent	SD	Range	N
Job change next year	7.89%	-	0–1	10861
Relocation next year	4.96%	-	0–1	28778
Life satisfaction	6.93	1.75	0–10	28778
Housing satisfaction	7.88	1.80	0–10	28720
Job satisfaction	6.95	1.93	0–10	10735
Balanced Effect	1.10	1.35	-4–4	19537
Perceived control	32.97	6.61	7–49	6820
Sex (female)	52.46%	-	0–1	28778
Age	50.21	17.65	17–99	28778
Age^2^	2832.46	1831.63	289–9801	28778

### Measures

#### Dependent variables

*Job change and relocation*. The SOEP contains annually the self-reported information on whether persons changed their job or relocated within the last year. Based on these variables, we constructed the binary variable *“job change”* which contained the information if the person changes the job next year (1) or not (0) and the binary variable *“relocation”* which contained the information if persons are relocating in the following year (1) or not (0).

#### Independent variables

*General Life Satisfaction*. In the SOEP, *life satisfaction* is measured annually with a single item [34, 36]. Respondents answer the item *“How satisfied are you with your life*, *all things considered*?*”* on a scale of 0 (Completely dissatisfied) to 10 (Completely satisfied).

*Domain-Specific Satisfaction*. Satisfaction with 11 different life domains has been measured annually since 2004. Domain satisfaction is measured with one item for each domain and on the same 11-point scale [36, 37]. We examined job satisfaction *(“How satisfied are you with your job*?*”)* and housing satisfaction *(“How satisfied are you with your housing*?*”)*.

*Affective Well-Being*. Since 2007, four items assess respondents’ affective well-being [36, 38]. The respondents are asked how often in the last four weeks (1 =“Very rarely” to 5 =“very often”) they felt “happy”, “angry”, “worried” and “sad”. Whereas the item “happy” measures positive experiences, the other three items measure negative experiences. Scale scores were computed by subtracting the average of the three negative items from the score on the positive item [34].

*Perceived Control*. In 2005, 2010, and 2015 respondents’ perceived controls was measured on a scale from 1 (strongly disagree) to 7 (strongly agree) with 7 items: "How my life goes depends on myself"; "What one achieves in life is mainly a question of luck or fate"; "Compared to others, I haven't achieved what I deserved"; "I often have the experience that others make decisions regarding my life"; "When I encounter difficulties, I have doubts about my abilities"; "The opportunities I have in life are determined by social conditions"; "I have little control over the things that happen in my life." The items were recoded, so that higher values correspond to greater feeling of control. Then the items were summed up to build an index (alpha = 0.70) [36, 39]. For the analysis, all three measurement points of perceived control were used for the estimation if they were available.

Control variables used in our study were age, age^2^, marital status (1 = married, living together, 2 = married, living separately, 3 = unmarried, 4 = divorced, 5 = widowed) education in years, net income, work hours, and sex (0 = male,1 = female) of the respondents. Age^2^ was added to control the curvilinear effects of age.

### Statistical analysis

We used multilevel modeling to adequately address the clustered data structure (person-year observations clustered in persons). Due to the binary character of our dependent variables, we estimated logistic multilevel models, or more precisely random-intercept logistic models. The covariates were grand-mean centered. In the supplemental material, we additionally report the results with standardized covariates (S2–S5 Tables). We used the statistics software Stata 14.2 and the command xtlogit for the estimation of the multilevel models. Xtlogit provides maximum likelihood estimation and uses adaptive quadrature to approximate the involved integrals [40]. As integration method for the random effects, we used the default mean and variance adaptive Gauss–Hermite quadrature. Listwise deletion was used.

To test our hypotheses, we estimated two sets of models. In the first set of models (Tables 2 and 3), we tested the main effects of domain satisfaction, life satisfaction and affective-well-being on relocation and job change in the following year. In the first models (Tables 2 and 3, Model 1), the events were predicted using solely domain satisfaction (and control variables). In the second models (Tables 2 and 3, Model 2), life satisfaction replaced domain satisfaction. The third models (Tables 2 and 3, Model 3) considered domain satisfaction and life satisfaction simultaneously as predictors. The fourth models (Tables 2 and 3, Model 4) added affective well-being as covariate.

**Table 2:** Main effects of cognitive well-being (CWB) and affective well-being (AWB) on relocation next year.

	Model (1)	Model (2)	Model (3)	Model (4)
	Only DS	Only LS	CWB	CWB+AWB
Domain satisfaction (DS)	0.837*** (0.012)		0.832*** (0.013)	0.835*** (0.016)
Life satisfaction (LS)		0.951** (0.016)	1.024 (0.019)	1.050 (0.029)
Affective well-being (AWB)				0.962 (0.033)
controls	Yes	Yes	Yes	Yes
Observations	28720	28778	28636	19537

Relocate next year

Odds ratios; covariates centered, standard errors in parentheses; control variables: sex, age, age^2^.

* *p* < 0.05

** *p* < 0.01

*** *p* < 0.001.

**Table 3:** Main effects of cognitive well-being (CWB) and affective well-being (AWB) on job change.

	Model (1)	Model (2)	Model (3)	Model (4)
	Only DS	Only LS	CWB	CWB+AWB
Domain satisfaction (DS)	0.791*** (0.016)		0.776*** (0.017)	0.792*** (0.021)
Life satisfaction (LS)		0.921** (0.024)	1.065* (0.031)	1.113** (0.045)
Affective well-being (AWB)				0.929 (0.043)
controls	Yes	Yes	Yes	Yes
Observations	10735	10861	10717	7137

Job change next year

Odds ratios; covariates centered, standard errors in parentheses; control variables: sex, age, age^2^.

* *p* < 0.05

** *p* < 0.01

*** *p* < 0.001.

In the second set of models (Tables 4 and 5), we tested the multiplicative effects of domain satisfaction and life satisfaction on life events (Tables 4 and 5, Model 1) and the moderating effects of perceived control (Tables 4 and 5, Model 2 and Model 3).

**Table 4:** Two-way interaction effects relocate next year.

	Model (1)	Model (2)	Model (3)
	DS*LS	DS*LoC	LS*LoC
Relocate next year			
Domain satisfaction (DS)	0.831*** (0.016)	0.836*** (0.024)	0.838*** (0.024)
Life Satisfaction (LS)	1.020 (0.030)	0.973 (0.036)	0.987 (0.038)
DS*LS	0.974** (0.009)		
Affective Well-Being (AWB)	0.964 (0.033)		
Locus of Control (LoC)		0.995 (0.010)	0.999 (0.010)
DS*LoC		0.993 (0.004)	
LS*LOC			1.003 (0.004)
controls	Yes	Yes	Yes
Observations	19537	6820	6820

Relocate next year

Odds ratios; covariates centered; control variables: sex, age, age^2^.

standard errors in parentheses

* *p* < 0.05

** *p* < 0.01

*** *p* < 0.001.

**Table 5:** Two-way interaction effects job change.

	Model (1)	Model (2)	Model (3)
	DS*LS	DS*LoC	LS*LoC
Domain satisfaction (DS)	0.788*** (0.021)	0.769*** (0.047)	0.766*** (0.047)
Life Satisfaction (LS)	1.110* (0.048)	1.029 (0.075)	1.031 (0.075)
DS*LS	0.998 (0.013)		
Affective Well-Being (AWB)	0.931 (0.044)		
Locus of Control (LoC)		1.016 (0.018)	1.018 (0.018)
DS*LoC		0.994 (0.007)	
LS*LOC			0.998 (0.009)
controls	Yes	Yes	Yes
Observations	7137	2037	2037

Job change next year

Odds Ratios, covariates centered, control variables: sex, age, age^2^.

standard errors in parentheses

* *p* < 0.05, ** *p* < 0.01

*** *p* < 0.001.

For a more intuitive interpretation of the interaction effects, we present plotted adjusted predictions at the means that represent the predicted probability of the occurrence of the event for individuals with a specific set of values while fixing the other covariates at their means [41].

## Results

Before we examined the three competing hypotheses on the interrelation of domain satisfaction and life satisfaction, we tested Hypotheses 1 to 3. Hypothesis 1, according to which domain satisfaction is negatively related to changes in the focal domain if examined in the absence of general life satisfaction, could be confirmed for both examined life events (relocation and job change): Domain satisfaction was negatively related to relocation (Table 2, Model 1, OR .837, *p* < .001) and to job change (Table 3, Model 1, OR = .791, *p* < .001) in the following year. We could also confirm hypothesis 2: As predicted, life satisfaction decreased the likelihood of relocation (Table 2, Model 2; OR = .951, *p* = .003) and job change (Table 3, Model 2, OR = .921, *p* = .001). The effects of life satisfaction were smaller than the effects of domain satisfaction, which is in line with the assumption that changes in the focal domain are more strongly related to domain satisfaction (see also S1 and S2 Tables for standardized estimates).

Examining domain satisfaction and life satisfaction simultaneously did not substantially change the effect of domain satisfaction on relocation (Table 2, Model 3, OR = .832, *p* < .001) and job change (Table 3, Model 3, OR = .776, *p* < .001), confirming hypothesis 3 according to which the negative effect of domain satisfaction on events in the focal domain does not change if life satisfaction is considered as an additional covariate.

Controlling for affective well-being did not change the coefficients of domain satisfaction and life satisfaction (see Model 4 in Tables 2 and 3).

Afterwards we tested the three competing hypotheses on the interrelation of domain satisfaction and life satisfaction (Hypotheses 4a-4c). Results mainly supported hypothesis 4c (a > 0), according to which the effect of life satisfaction on events in the focal domain becomes positive if domain satisfaction is taken into account: For both domains, the signs of the effects of life satisfaction on life events flipped from negative to positive if domain satisfaction was controlled for. Note, however, that only the effect of life satisfaction on job change in the following year was significant (Table 3, Model 3, OR = 1.065, *p* = .031), but not the effect of life satisfaction on relocation (Table 2, Model 3, OR = 1.024, *p* = .205). Therefore, we only found mixed evidence for hypothesis 4c (a > 0) and partial support for hypothesis 4a (a = 0) that presumed that only domain satisfaction plays a role for change in the focal domain and not life satisfaction (a = 0). Hypothesis 4b which presumed that life satisfaction decreases the likelihood of events in the focal domain (a < 0) could clearly be rejected.

**Fig 2:**
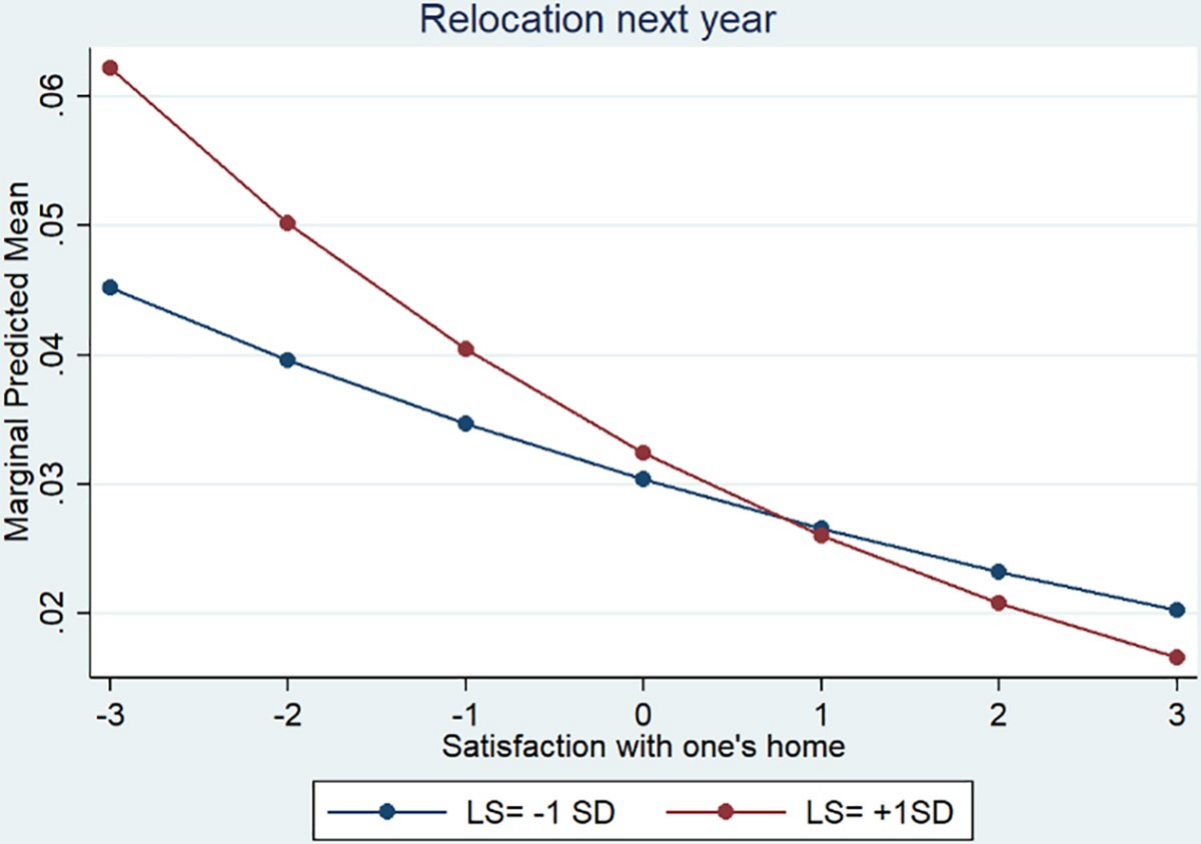
Adjusted predictions, relocate next year.

Additionally, we explored the interaction of life satisfaction and domain satisfaction. The interaction effects were small and negative for both domains, but whereas for relocation the interaction effect was significant (Table 4, Model 1, OR = .974, *p* = .004), this was not the case for job change (Table 5, Model 1, OR = .998, *p* = .877). A plot of the interaction effect of life satisfaction and domain satisfaction on relocation (see Fig 2) showed that especially for persons with high life satisfaction and low domain satisfaction the likelihood was higher to relocate in the following year. This result is consistent with hypothesis 4c (a > 0) which assumed that people only strive for change if they possess sufficient resources that are indicated through high life satisfaction. Note that a significant interaction effect could be found for relocation only, so the pattern differed across the two domains.

Finally, we tested the moderating effects of perceived control (Hypothesis 5 and 6). Somewhat unexpectedly, perceived control moderated neither the effect of domain satisfaction (Table 4, Model 2 and Model 3) nor the effect of life satisfaction on life events in the focal domain (Table 5, Model 2 and 3). Therefore, these results did not support our hypotheses on the moderating effect of perceived control.

### Sensitivity analyses

To address the issue that sociodemographic variables might have affected our results, we ran analyses that controlled for the following variables: marital status, working hours per week, education in years, and net income. To take into account that the events of divorce or separation might had an effect on the events of relocation or job change, we also added the variables divorce next year and separation next year to the models. For job change, only marginal changes in the coefficients could be observed (S6 and S8 Tables). Similarly, the interaction effects for relocation did not change substantially (S7 Table). However, there were some substantial changes with respect to the main effects for relocation. The size of the isolated effect of life satisfaction on relocation changed only slightly but became non-significant (S5 Table, Model 2, OR = .973, *p* = .241). Other changes could be observed in respect to the joint effects of domain satisfaction and life satisfaction on relocation (S5 Table, Model 3 and 4). The effect sizes remain almost the same, but the effects of life satisfaction became significant (S5 Table, Model 3, OR = 1.060, *p* = .020, Model 4, OR = 1.087, *p* = .022). Therefore, the effects are not robust and should be interpreted with caution.

To test if multicollinearity between our main predictors (life satisfaction, domain satisfaction, affective well-being and perceived control) is a problem, we estimated pooled logistic regression models with clustered standard errors within persons and calculated the variance inflation factors (VIFs) using the Stata program collin [42] (see S9 and S10 Tables). A VIF above 4 or 10 is commonly used as threshold to indicate multicollinearity [43]. Our results show VIFs ranging from 1.14 to 1.63 (see S9 and S10 Tables). This is well beyond the common used thresholds, indicating no problems with multicollinearity.

## Discussion

When and why do people change their jobs, their homes, or their partners? We argued that people actively initiate these changes because they are dissatisfied with their life circumstances and try to overcome their dissatisfaction by initiating changes in their life domains. According to the motivational theory of the life-span development, life domains are interdependent [44]. Taking this assumption seriously implies that life domains should not be studied in isolation. In this study, we therefore considered life satisfaction (reflecting satisfaction with multiple life domains) in addition to satisfaction with the focal domain (job satisfaction and housing satisfaction) together in one model to predict changes in the focal domain (job change and relocation).

At a first glance, our results appeared to confirm former research that studied domain satisfaction and life satisfaction as predictors of life events in isolation [1, 3–9]. Consistent with prior research, low job satisfaction and low satisfaction with housing increased the probability of job change and relocation if tested in isolation [1, 3–7, 10]. However, the picture changed when examining domain satisfaction and life satisfaction simultaneously in one model: Low domain satisfaction remained a significant predictor of life events, but the effect of life satisfaction either flipped from negative to positive or became non-significant.

These results support the third of our three competing hypothesis that presumed that people only strive for change in their life if they perceive that they possess the necessary resources (indicated through high life satisfaction). These results underpin the interdependence of domains as stated in the motivational theory of life span development [44] and can be seen as a call for further research to take into account not only general life satisfaction (indicating satisfaction with multiple life domains) but also satisfaction with the focal domain.

Our results may appear to imply that high life satisfaction is always desirable. But this is not always the case. In fact, our model also makes a case for potentially beneficial effects of lower satisfaction. Specifically, we assume that lower levels of satisfaction may motivate people to increase their levels of satisfaction (with a particular domain or with life in general) by improving their life circumstances. Indeed, this assumption is consistent with previous research that showed that people who are most successful in education and people with a high income do not report the highest amount of life satisfaction [45, 46]. Complete satisfaction, in contrast, “might prevent individuals from energetically pursuing change in achievement domains such as education and income” [47].

Other implications for further research concern the constructs of affective well-being and perceived control. In our study, affective well-being did not play a role in predicting life events after accounting for cognitive well-being (domain satisfaction and life satisfaction). This is in contrast to common motivational theories that highlight the role of affect in motivation [29, 30], but confirms the findings of previous studies that cognitive well-being is more strongly related to life events than affective well-being [48]. Somewhat surprisingly, perceived control did not play a role as moderating variable. We suggest that this could be due to perceived control being measured on a general level and not domain specific. Since we could observe that domain satisfaction in the focal domains is more strongly related to life events, this could also have been the case for perceived control if it had been measured separately for each domain. The same is also true for affective well-being, which is also not measured domain specific.

Another limitation of our study was that job change and relocation are complex human experiences and our paper only addresses a specific set of possible predictors. We addressed this point in the subsection Sensitivity Analysis. We added more control variables that may have an impact on the outcomes to our statistical models. Results showed that our main findings were robust for job change, but not for relocation (see Sensitivity Analyses for details).

A limitation of the analyzed data is that we cannot say for sure that the individuals actively initiated the changes within the life domains. This limitation could be addressed in future studies that include annual measures asking participants directly whether they aim to change their life circumstances in the different life domains. Another way to address this limitation could be the use of experimental data. For instance, previous research that used experimental data with manipulated life satisfaction, could demonstrate that desire for change was significantly higher for people with low life satisfaction than for people high in life satisfaction [11].

A further limitation of our study was that the analyses were limited to two life domains. We considered these domains because they are to a certain degree controllable and are not too infrequent in the data we analyzed (see Table 1). Therefore, even if we cannot generalize our results to all kinds of life domains, our results can give important hints to the motivational consequences of subjective well-being for life events. Besides these limitations, we only have annual measures of our variables and therefore only get a limited picture of individual life courses. Despite these limitations, the data we used also had some important advantages: First, we could analyze large samples (*N*s between 2,037 and 28,778), Second, the panel data structure offered us the opportunity to predict life events prospectively by independent variables that were measured one year before the event took place (or not). These advantages could also be used for examining the dynamic interplay of life satisfaction and domain satisfaction. How these constructs influence each other over time may be an interesting question for further research.

In conclusion, future studies should not only focus on domain satisfaction, and consider additionally life satisfaction, since we found evidence that life satisfaction, may play a role as resource that enables people to strive for change.

## Supporting information

S1 Table Main effects of cognitive well-being and affective well-being on relocation next year, standardized covariates. (DOCX)

S2 Table Main effects of cognitive well-being and affective well-being on job change, standardized covariates. (DOCX)

S3 Table Two-way interaction effects relocate next year, standardized covariates. (DOCX)

S4 Table Two-way interaction effects job change, standardized covariates. (DOCX)

S5 Table Main effects of cognitive well-being and affective well-being on relocation next year with further control variables. (DOCX)

S6 Table Main effects of cognitive well-being and affective well-being on job change with further control variables. (DOCX)

S7 Table Two-way interaction effects relocate next year with further control variables. (DOCX)

S8 Table Two-way interaction effects job change with further control variables. (DOCX)

S9 Table Variance inflation factors (VIFs) for main predictors of relocation. (DOCX)

S10 Table Variance inflation factors (VIFs) for main predictors of job change. (DOCX)
