# Supplementary material for: The interplay of domain-and life satisfaction in predicting life events
Source: PLoS One. 2020 Sep 17;15(9):e0238992. doi: 10.1371/journal.pone.0238992 (PMC7498007; doi:10.1371/journal.pone.0238992)
Supplement: S1 Table — (DOCX) [file pone.0238992.s001.docx]

*S1 Table.* Main effects of cognitive well-being and affective well-being on relocation next year, standardized covariates

Relocate next year

|  | Model (1) | Model (2) | Model (3) | Model (4) |
| --- | --- | --- | --- | --- |
|  | Only DS | Only LS | CWB | CWB+AWB |
|  |  |  |  |  |
| Domain satisfaction (DS) | 0.727^***^ (0.018) |  | 0.719^***^ (0.020) | 0.724^***^ (0.025) |
| Life satisfaction (LS) |  | 0.916^**^ (0.0267) | 1.042 (0.034) | 1.089 (0.053) |
| Affective well-being (AWB) |  |  |  | 0.949 (0.043) |
| controls | Yes | Yes | Yes | Yes |
| Observations | 28720 | 28778 | 28636 | 19537 |

*Notes.* Odds ratios; covariates standardized, standard errors in parentheses; Control variables: sex, age, age²

^*^ *p* < 0.05, ^**^ *p* < 0.01, ^***^ *p* < 0.001
