# Supplementary material for: The interplay of domain-and life satisfaction in predicting life events
Source: PLoS One. 2020 Sep 17;15(9):e0238992. doi: 10.1371/journal.pone.0238992 (PMC7498007; doi:10.1371/journal.pone.0238992)
Supplement: S2 Table — (DOCX) [file pone.0238992.s002.docx]

*S2 Table.* Main effects of cognitive well-being and affective well-being on job change, standardized covariates

Job change next year

|  | Model (1) | Model (2) | Model (3) | Model (4) |
| --- | --- | --- | --- | --- |
|  | Only DS | Only LS | CWB | CWB+AWB |
|  |  |  |  |  |
| Domain satisfaction (DS) | 0.632^***^ (0.025) |  | 0.609^***^ (0.027) | 0.634^***^ (0.021) |
| Life satisfaction (LS) |  | 0.878^**^ (0.035) | 1.105^*^ (0.051) | 1.184^**^ (0.077) |
| Affective well-being (AWB) |  |  |  | 0.910 (0.055) |
| controls | Yes | Yes | Yes | Yes |
| Observations | 10735 | 10861 | 10717 | 7137 |

*Notes.* Odds ratios; standardized covariates, standard errors in parentheses; Control variables: sex, age, age²

^*^ *p* < 0.05, ^**^ *p* < 0.01, ^***^ *p* < 0.001
