# Supplementary material for: The interplay of domain-and life satisfaction in predicting life events
Source: PLoS One. 2020 Sep 17;15(9):e0238992. doi: 10.1371/journal.pone.0238992 (PMC7498007; doi:10.1371/journal.pone.0238992)
Supplement: S3 Table — (DOCX) [file pone.0238992.s003.docx]

*S3 Table.* Two-way interaction effects relocate next year, standardized covariates

Relocate next year

|  | Model (1) | Model (2) | Model (3) |
| --- | --- | --- | --- |
|  | DS*LS | DS*LoC | LS*LoC |
| Relocate next year |  |  |  |
| Domain satisfaction (DS) | 0.717^***^ (0.024) | 0.724^***^ (0.038) | 0.729^***^ (0.038) |
| Life Satisfaction (LS) | 1.035 (0.053) | 0.954 (0.061) | 0.977 (0.066) |
| DS*LS | 0.921^**^ (0.026) |  |  |
| Affective Well-Being (AWB) | 0.951 (0.043) |  |  |
| Perceived Control (PC) |  | 0.965 (0.064) | 0.996 (0.065) |
| DS*PC |  | 0.922 (0.043) |  |
| LS*PC |  |  | 1.038 (0.053) |
| controls | Yes | Yes | Yes |
| Observations | 19537 | 6820 | 6820 |

*Notes.* Odds ratios; covariates centered; Control variables: sex, age, age²
standard errors in parentheses; ^*^ *p* < 0.05, ^**^ *p* < 0.01, ^***^ *p* < 0.001
