# Supplementary material for: The interplay of domain-and life satisfaction in predicting life events
Source: PLoS One. 2020 Sep 17;15(9):e0238992. doi: 10.1371/journal.pone.0238992 (PMC7498007; doi:10.1371/journal.pone.0238992)
Supplement: S4 Table — (DOCX) [file pone.0238992.s004.docx]

*S4 Table.* Two-way interaction effects job change, standardized covariates

Job change next year

|  | Model (1) | Model (2) | Model (3) |
| --- | --- | --- | --- |
|  | DS*LS | DS*LoC | LS*LoC |
|  |  |  |  |
| Domain satisfaction (DS) | 0.628*** (0.033) | 0.594*** (0.071) | 0.594*** (0.071) |
| Life Satisfaction (LS) | 1.179* (0.081) | 1.045 (0.075) | 1.047 (0.123) |
| DS*LS | 0.994 (0.041) |  |  |
| Affective Well-Being (AWB) | 0.911 (0.055) |  |  |
| Perceived Control (PC) |  | 1.103 (0.126) | 1.118 (0.126) |
| DS*PC |  | 0.933 (0.086) |  |
| LS*PC |  |  | 0.981 (0.089) |
| controls | Yes | Yes | Yes |
| Observations | 7137 | 2037 | 2037 |

*Notes.* Odds ratios; covariates centered; Control variables: sex, age, age²
standard errors in parentheses; ^*^ *p* < 0.05, ^**^ *p* < 0.01, ^***^ *p* < 0.001
