# Supplementary material for: The interplay of domain-and life satisfaction in predicting life events
Source: PLoS One. 2020 Sep 17;15(9):e0238992. doi: 10.1371/journal.pone.0238992 (PMC7498007; doi:10.1371/journal.pone.0238992)
Supplement: S5 Table — (DOCX) [file pone.0238992.s005.docx]

*S5 Table.* Main effects of cognitive well-being and affective well-being on relocation next year with further control variables

Relocate next year

|  | Model (1) | Model (2) | Model (3) | Model (4) |
| --- | --- | --- | --- | --- |
|  | Only DS | Only LS | CWB | CWB+AWB |
|  |  |  |  |  |
| Domain satisfaction (DS) | 0.828^***^ (0.015) |  | 0.815^***^ (0.016) | 0.812^***^ (0.019) |
| Life satisfaction (LS) |  | 0.973 (0.022) | 1.060^*^ (0.026) | 1.087^*^ (0.040) |
| Affective well-being (AWB) |  |  |  | 0.965 (0.041) |
| Controls |  |  |  |  |
| Sex | 1.001 (0.086) | 0.981 (0.084) | 1.009 (0.086) | 1.070 (0.110) |
| Age (centered) | 0.984 (0.025) | 0.995 (0.025) | 0.991 (0.025) | 0.994 (0.031) |
| Age² (centered) | 0.999 (0.000) | 0.999^*^ (0.000) | 0.999^*^ (0.000) | 0.999 (0.000) |
| Education in years | 1.024 (0.016) | 1.022 (0.016) | 1.023 (0.016) | 1.027 (0.020) |
| Net income | 1.000^*^ (0.000) | 1.000^**^ (0.000) | 1.000^*^ (0.000) | 1.000^*^ (0.000) |
| Marital status 1. married, living together (ref.) |  |  |  |  |
| 2. married, living separately | 3.187^***^ (0.558) | 3.476^***^ (0.606) | 3.271^***^ (0.573) | 3.562^***^ (0.745) |
| 3. unmarried | 1.382^**^ (0.146) | 1.453^***^ (0.154) | 1.388^**^ (0.146) | 1.261 (0.163) |
| 4. divorced | 2.435^***^ (0.304) | 2.611^***^ (0.326) | 2.420^***^ (0.303) | 2.706^***^ (0.395) |
| 5. widowed | 1.455 (0.698) | 1.507 (0.720) | 1.450 (0.695) | 1.420 (0.858) |
| Weekly work hours | 1.007 (0.004) | 1.007^*^ (0.004) | 1.007^*^ (0.004) | 1.010^*^ (0.004) |
| Separation next year | 0.634 (0.284) | 0.658 (0.294) | 0.650 (0.291) | 0.727 (0.395) |
| Divorce next year | 0.998 (0.788) | 1.073 (0.841) | 1.053 (0.827) | N/A |
| Observations | 15084 | 15102 | 15056 | 10371 |

*Notes.* Odds ratios; DS, LS, and AWB are centered, standard errors in parentheses;

* p < 0.05, ** p < 0.01, *** p < 0.001*
