# Supplementary material for: The interplay of domain-and life satisfaction in predicting life events
Source: PLoS One. 2020 Sep 17;15(9):e0238992. doi: 10.1371/journal.pone.0238992 (PMC7498007; doi:10.1371/journal.pone.0238992)
Supplement: S6 Table — (DOCX) [file pone.0238992.s006.docx]

*S6 Table.* Main effects of cognitive well-being and affective well-being on job change with further control variables

Job change next year

|  | Model (1) | Model (2) | Model (3) | Model (4) |
| --- | --- | --- | --- | --- |
|  | Only DS | Only LS | CWB | CWB+AWB |
|  |  |  |  |  |
| Domain satisfaction (DS) | 0.785^***^ (0.017) |  | 0.768^***^ (0.018) | 0.778^***^ (0.022) |
| Life satisfaction (LS) |  | 0.930^**^ (0.026) | 1.077^*^ (0.034) | 1.120^**^ (0.049) |
| Affective well-being (AWB) |  |  |  | 0.948 (0.047) |
| Controls |  |  |  |  |
| Sex | 0.737^**^ (0.083) | 0.756^*^ (0.082) | 0.738^**^ (0.083) | 0.771^*^ (0.100) |
| Age (centered) | 0.819^***^ (0.025) | 0.845^***^ (0.024) | 0.822^***^ (0.025) | 0.838^***^ (0.029) |
| Age² (centered) | 1.002^***^ (0.000) | 1.001^***^ (0.000) | 1.002^***^ (0.000) | 1.001^**^ (0.000) |
| Education in years | 1.141^***^ (0.023) | 1.135^***^ (0.023) | 1.139^***^ (0.023) | 1.125^***^ (0.026) |
| Net income | 1.000^***^ (0.000) | 1.000^***^ (0.000) | 1.000^***^ (0.000) | 1.000^***^ (0.000) |
| Marital status 1. married, living together (ref.) |  |  |  |  |
| 2. married, living separately | 1.046 (0.295) | 0.990 (0.274) | 1.070 (0.302) | 1.005 (0.339) |
| 3. unmarried | 0.880 (0.118) | 0.931 (0.122) | 0.884 (0.119) | 0.817 (0.128) |
| 4. divorced | 1.301 (0.228) | 1.385 (0.236) | 1.324 (0.232) | 1.193 (0.240) |
| 5. widowed | 1.917 (0.967) | 1.918 (0.930) | 1.932 (0.975) | 1.601 (0.950) |
| Weekly work hours | 0.996 (0.005) | 0.999 (0.005) | 0.996 (0.005) | 0.996 (0.006) |
| Separation next year | 1.906 (1.013) | 1.527 (0.785) | 1.981 (1.058) | 2.139 (1.409) |
| Divorce next year | 1.331 (1.161) | 1.468 (1.253) | 1.390 (1.204) | N/A |
| Observations | 10069 | 10167 | 10055 | 6667 |

*Notes.* Odds ratios; DS, LS, and AWB are centered, standard errors in parentheses;

* p < 0.05, ** p < 0.01, *** p < 0.001*
