# Supplementary material for: The interplay of domain-and life satisfaction in predicting life events
Source: PLoS One. 2020 Sep 17;15(9):e0238992. doi: 10.1371/journal.pone.0238992 (PMC7498007; doi:10.1371/journal.pone.0238992)
Supplement: S7 Table — (DOCX) [file pone.0238992.s007.docx]

*S7 Table.* Two-way interaction effects relocate next year with further control variables

Relocate next year

|  | Model (1) | Model (2) | Model (3) |
| --- | --- | --- | --- |
|  | DS*LS | DS*LoC | LS*LoC |
|  |  |  |  |
| Domain satisfaction (DS) | 0.810^***^ (0.019) | 0.822^***^ (0.032) | 0.820^***^ (0.032) |
| Life satisfaction (LS) | 1.049 (0.041) | 1.023 (0.052) | 1.043 (0.055) |
| DS*LS | 0.971^*^ (0.012) |  |  |
| Affective well-being (AWB) | 0.968 (0.041) |  |  |
| Perceived Control (PC) |  | 1.000 (0.013) | 1.004 (0.013) |
| DS*PC |  | 0.994 (0.005) |  |
| LS*PC |  |  | 1.009 (0.006) |
| Controls |  |  |  |
| Sex | 1.074 (0.110) | 1.057 (0.177) | 1.039 (0.174) |
| Age (centered) | 0.993 (0.031) | 0.947 (0.045) | 0.953 (0.046) |
| Age² (centered) | 0.999 (0.000) | 1.000 (0.001) | 1.000 (0.001) |
| Education in years | 1.026 (0.020) | 0.971 (0.032) | 0.971 (0.032) |
| Net income | 1.000^*^ (0.000) | 1.000^**^ (0.000) | 1.000^**^ (0.000) |
| Marital status 1. married, living together (ref.) |  |  |  |
| 2. married, living separately | 3.559^***^ (0.744) | 3.009^**^ (1.119) | 3.048^**^ (1.132) |
| 3. unmarried | 1.255 (0.162) | 1.298 (0.280) | 1.322 (0.284) |
| 4. divorced | 2.660^***^ (0.389) | 1.957^**^ (0.503) | 1.958^**^ (0.502) |
| 5. widowed | 1.367 (0.826) | 3.200 (2.031) | 3.182 (2.020) |
| Weekly work hours | 1.010^*^ (0.004) | 1.022^**^ (0.007) | 1.023^**^ (0.007) |
| Separation next year | 0.741 (0.401) | 0.468 (0.491) | 0.447 (0.469) |
| Divorce next year | N/A | 2.326 (2.706) | 1.858 (2.216) |
| Observations | 10371 | 3546 | 3546 |

*Notes.* Odds ratios; DS, LS, PC and AWB are centered, standard errors in parentheses;

* p < 0.05, ** p < 0.01, *** p < 0.001*
