# Supplementary material for: The interplay of domain-and life satisfaction in predicting life events
Source: PLoS One. 2020 Sep 17;15(9):e0238992. doi: 10.1371/journal.pone.0238992 (PMC7498007; doi:10.1371/journal.pone.0238992)
Supplement: S8 Table — (DOCX) [file pone.0238992.s008.docx]

*S8 Table.* Two-way interaction effects job change with further control variables
Job change next year

|  | Model (1) | Model (2) | Model (3) |
| --- | --- | --- | --- |
|  | DS*LS | DS*LOC | LS*LoC |
|  |  |  |  |
| Domain satisfaction (DS) | 0.779^***^ (0.022) | 0.754^***^ (0.051) | 0.753^***^ (0.051) |
| Life satisfaction (LS) | 1.105^*^ (0.050) | 1.039 (0.080) | 1.038 (0.080) |
| DS*LS | 0.987 (0.014) |  |  |
| Affective well-being (AWB) | 0.950 (0.047) |  |  |
| Perceived Control (PC) |  | 1.014 (0.020) | 1.016 (0.020) |
| DS*PC |  | 0.996 (0.008) |  |
| LS*PC |  |  | 0.997 (0.010) |
| Controls |  |  |  |
| Sex | 0.769^*^ (0.100) | 0.666 (0.178) | 0.670 (0.179) |
| Age (centered) | 0.837^***^ (0.029) | 0.725^***^ (0.059) | 0.724^***^ (0.059) |
| Age² (centered) | 1.001^**^ (0.000) | 1.003^***^ (0.001) | 1.003^***^ (0.001) |
| Education in years | 1.124^***^ (0.026) | 1.181^***^ (0.059) | 1.180^***^ (0.059) |
| Net income | 1.000^***^ (0.000) | 1.000^*^ (0.000) | 1.000^*^ (0.000) |
| Marital status 1. married, living together (ref.) |  |  |  |
| 2. married, living separately | 1.000 (0.338) | 1.480 (0.934) | 1.476 (0.932) |
| 3. unmarried | 0.820 (0.129) | 0.995 (0.325) | 0.988 (0.323) |
| 4. divorced | 1.195 (0.240) | 1.525 (0.619) | 1.514 (0.614) |
| 5. widowed | 1.598 (0.948) | 1.066 (1.287) | 1.066 (1.285) |
| Weekly work hours | 0.996 (0.006) | 0.994 (0.012) | 0.994 (0.012) |
| Separation next year | 2.113 (1.391) | 0.525 (0.781) | 0.530 (0.788) |
| Divorce next year | N/A | 8.426 (13.437) | 9.127 (14.643) |
| Observations | 6667 | 1920 | 1920 |

*Notes.* Odds ratios; DS, LS, PC and AWB are centered, standard errors in parentheses;

* p < 0.05, ** p < 0.01, *** p < 0.001*
