# Supplementary material for: The interplay of domain-and life satisfaction in predicting life events
Source: PLoS One. 2020 Sep 17;15(9):e0238992. doi: 10.1371/journal.pone.0238992 (PMC7498007; doi:10.1371/journal.pone.0238992)
Supplement: S9 Table — (DOCX) [file pone.0238992.s009.docx]

*S9 Table.* Variance Inflation Factors (VIFs) for main predictors of relocation

| Relocate next year | Model 1 | Model 2 | Model 3 |
| --- | --- | --- | --- |
|  | CWB (DS+LS) | CWB + AWB | CWB + PC |
| Domain satisfaction (DS) | 1.18 | 1.19 | 1.17 |
| Life Satisfaction (LS) | 1.18 | 1.61 | 1.28 |
| Affective Well-Being (AWB) | - | 1.52 | - |
| Perceived Control (PC) | - | - | 1.15 |
| Observations | 28636 | 19537 | 6820 |

*Notes.* VIFs are calculated based on pooled logistic regression models with clustered standard-errors within persons. Control variables: sex, age, age²
